# Effect of Time‐Restricted Eating on Metabolic Adaptation in Adults With Severe Obesity During Early Phase of Weight Loss

**DOI:** 10.1111/jhn.70293

**Published:** 2026-06-11

**Authors:** Tatiana Almeida de Moraes Campos, Raiane Lira Freitas Brasil, Flávia Fioruci Bezerra

**Affiliations:** ^1^ Nutrition Institute State University of Rio de Janeiro Rio de Janeiro Brazil

**Keywords:** body composition, caloric restriction, intermittent fasting, resting metabolic rate, weight loss

## Abstract

**Objective:**

To compare metabolic adaptations at the early phase of weight loss in individuals with severe obesity assigned to two strategies of energy restriction: continuous or in combination with time‐restricted eating (TRE).

**Methods:**

100 participants (> 40 kg/m^2^; 20–60 years) were randomized into two groups: continuous energy restriction (CER, *n* = 42) and TRE combined with energy restriction (TRE + ER; eating window: 10am‐6pm). Resting metabolic rate (RMR) by indirect calorimetry, body composition by dual‐energy X‐ray absorptiometry (DXA), serum hormones, and appetite feelings were evaluated at baseline and after 1 week of prescribed diet at 60% the total energy expenditure. Early adaptive thermogenesis (AT) was calculated using measured and predicted RMR. Data were evaluated using ANCOVA and linear mixed‐effects model.

**Results:**

After adjustment for RMR at baseline and body composition, RMR after 1 week was similar between groups (β_group = 47 ± 32 kcal/day; *p* = 0.148). Early AT was significantly lower in the TRE + ER group (−13 ± 160 kcal *vs*. −83 ± 150 kcal; *p* = 0.038). After a week of energy restriction, leptin, insulin, and TSH concentrations decreased in both groups (time effect, *p* < 0.05) with no group x time interaction. The feeling of hunger increased after 1 week only in the CER group (*p* = 0.048).

**Conclusion:**

Results suggest that TRE modestly attenuate early AT, but this effect does not translate into differences in RMR after 1 week. Hormonal and appetite responses were also not substantially affected by TRE. Further studies are needed to determine whether the modest early effects of TRE translate into clinically meaningful long‐term benefits for weight management.

## Summary

- Most adults with severe obesity showed adaptive thermogenesis (AT) after a week.
- TRE modestly attenuated early AT without affecting resting metabolic rate.
- Hormonal and appetite responses were not substantially affected by TRE.

## 1 Introduction

Early metabolic adaptations occur within the first days of energy restriction (ER) and are characterized by coordinated physiological and endocrine responses [1] that aim to conserve energy in situations of limited food intake [2, 3, 4, 5]. The initial phase of weight loss is marked by a decline in plasma insulin levels and a concomitant increase in glucagon secretion. These changes are accompanied by decreases in circulating leptin and by adaptive thermogenesis (AT) (i.e., a reduction in resting metabolic rate ‐RMR‐ i.e. disproportionate to the initial changes in body composition) [1, 6]. There is evidence that AT is fully established within 1 week of energy restriction (early AT), with no further changes in subsequent weeks [7, 8]. Early AT appears to predict body mass and fat mass (FM) loss after several weeks of energy restriction [8, 9] suggesting that it may impair the effectiveness of nutritional treatments aiming for weight loss. This is of special concern in the context of severe obesity, which is projected to increase from 2% to 5% in men and from 5% to 10% in women globally between 2010 and 2030, leading to more serious health consequences, and higher public health costs [10, 11, 12, 13].

Time‐restricted eating (TRE) is a dietary strategy that restricts eating window up to 10 h/day with no explicit limit on energy intake [14, 15]. TRE appears to promote synchrony between the central and peripheral circadian clocks, which helps reduce body weight [16, 17] though the exact mechanisms are still debated. Some studies suggest that TRE increases energy expenditure [18, 19], while others propose that weight loss results from decreased appetite and/or energy intake [16, 17, 20]. Studies evaluating the effect of TRE in combination with energy restriction on weight loss have adopted different eating windows and intervention length, usually over 3 weeks. In comparison with continuous energy restriction, strategies combining TRE resulted in more pronounced FM loss [21, 22, 23] and reduced leptin [24], with no effects on appetite feelings [25] and RMR [26]. Although absolute RMR does not appear to be affected, its disproportionate decline relative to changes in body composition (i.e., AT) in response to TRE has not been investigated. We hypothesized that TRE may optimize weight loss by attenuating the early metabolic adaptation induced by energy restriction. Therefore, this randomized controlled trial aims to compare metabolic adaptations at the early phase of weight loss in individuals with severe obesity assigned to two strategies of energy restriction: continuous or in combination with TRE.

## 2 Methods

This is a sub‐study of the randomized controlled parallel‐arm trial “Metabolic Adaptations during Caloric Restriction in Obesity” (MACRO‐ REBEC identifier: RBR‐45fpgqh). The trial was approved by the Institutional Ethics Committee (Hospital Universitário Pedro Ernesto/UERJ n° 7.075.010) and all participants read and signed the informed consent form to participate in the study.

### 2.1 Participants

Participants were recruited through widespread publicity on social media and publicity posters. Eligible participants were adults (20–60 years old) with severe obesity (BMI ≥ 40 kg/m^2^). Interested individuals completed an electronic form and were not included if they had the following conditions that could have interfered with body composition or RMR: pregnancy; lactation; use of illicit drugs; infectious or oncological diseases; thyroid disorders; heart, kidney, liver, or lung insufficiency; use of medications that alter energy metabolism (corticosteroids, beta‐blockers, sulfonylureas, GLP‐1 agonists, DPP4 inhibitors, and SGLT2 inhibitors); use of medications for obesity treatment (serotonergic, noradrenergic, and inhibitors of intestinal lipid absorption); individuals who underwent ER, had > 10% body mass change in 6 months, or had bariatric surgery.

The primary outcome in the present study is the difference in early AT between the intervention groups. Since no previous studies have compared AT between groups in the early phase of weight loss, the statistical power calculation was based on published data from the MATADOR trial [27], which reported a difference in AT between groups after 16 weeks of intervention (−145 ± 121 vs. −60 ± 121 kcal). With the MACRO sample size (*n* = 50 across two groups) and based on previous study, the estimated minimum detectable differences in AT between the groups was 68 kcal, assuming a 95% CI and 80% statistical power. The sample size of MACRO provided a statistical power of 94%.

### 2.2 Study Design

Eligible individuals were invited to attend a pre‐study visit at the Nutrition Clinic for outpatients' care at the Piquet Carneiro Policlinic of State University of Rio de Janeiro (UERJ) to complete an interview that included socioeconomic, general health, eating habits and anthropometric data. Participants were scheduled for their first visit to the Nutritional Assessment Laboratory to begin the study.

At baseline, participants were randomly assigned to either follow an energy restriction diet combined with TRE (TRE + ER group) or a continuous energy restriction diet (CER group). Random assignment (1:1) used permuted blocks of four via randomizer.org. Due to the nature of the intervention, neither the researchers nor the participants were blinded. The first follow‐up occurred after 1 week to assess early metabolic adaptation to energy restriction.

At both timepoints (baseline and after 1 week), participants were instructed to visit the lab for data collection after fasting for 10 to 16 h overnight. Additionally. they were instructed to avoid (24 h) smoking, alcohol, vigorous exercise, and ensure 6–8 h sleep before measurements. A questionnaire was administered to confirm adherence to this protocol. Blood samples, body composition, RMR, and appetite feelings data were collected.

### 2.3 Nutritional Intervention

The total energy expenditure (TEE) was calculated based on measured RMR and the physical activity factor (PAF). The prescription of energy restriction in both groups considered 60% of the calculated TEE (TEE_60%_) [28]. The dietary prescription was designed to maintain a normoprotein (0.8 to 1.0 g/kg/d), normoglycidic (50% to 60%), and normolipidic (25% to 30%) diet using Webdiet software (© 2024 WebDiet Health Manager). The food choices for the diet were tailored to the eating habits of each participant. All participants in the TRE + ER group were instructed to adhere to an 8‐h eating window (from 10 am to 6 pm).

The energy restriction adherence was determined by the difference between the actual daily energy (kcal) lost and the expected daily energy lost (daily TEE‐ energy prescribed). The actual daily energy (kcal) lost was calculated as:

− In the case of FFM loss: [(9.3* ΔFM(g)) + ((1.1 *‐ΔFFM(g))/days in energy restriction)]
− In the case of FFM gain: [(9.3 × ΔFM (g)) + (1.8 × ΔFFM (g))/days in energy restriction]

Negative values indicate a smaller than expected weight loss, considering the energy restriction performed. Adherence to energy restriction is expressed as a percentage [2, 29].

### 2.4 RMR and Total Energy Expenditure

RMR was measured by indirect calorimetry (Vmax Encore 29, Sensormedics, Palm Springs, CA) with the subject awake in supine position, using a canopy in a quiet, dark, noiseless, thermoneutral room between 7:00 and 9:30 am. The participants rested for 15 min before starting the measurement. After flow and gas calibration, the volume of oxygen consumed (VO_2_) and carbon dioxide produced (VCO_2_) were measured for at least 15 min, or as long as necessary to obtain steady state (CV < 10% for at least 5 min). The coefficients of variation of VO_2_ and VCO_2_ were < 6.5%. The first 5 min were excluded from the calculations. VO_2_ and VCO_2_ were expressed in kcal/day according to Weir's equation (1949) [30].

Physical activity level was estimated at baseline using International Physical Activity Questionnaire (IPAQ) data and categorized according to the physical activity factor (PAF) criteria proposed by IOM (2005) [31]: PAF 1.0–1.4 (inactive/irregularly active B), 1.4–1.6 (irregularly active A), 1.6–1.9 (active), and 1.9–2.5 (very active). TEE was calculated by multiplying the measured RMR by the estimated PAF.

### 2.5 Early Adaptive Thermogenesis

As previously suggested [32] predicted RMR (RMRp) was derived from the following equation using baseline data of the present study:

RMRp(kcal/d)=[(16.168*FFM(kg))+(−0.039*FM(kg))+(−3.350*AGE(years))–(43.406*sex)+764.159]

$$\text{Sex}\left(\text{Women}\;=1,\text{Men}=0\right),R^{2}\text{adjusted}=0.58;p<0.0001$$

Early AT at the RMR level was determined by calculating the difference between the measured (RMRm) and RMRp, according to Nunes et al. [32]: [(RMRm ‐ RMRp (intervention)] – [RMRm ‐ RMRp (baseline)].

### 2.6 Body Composition

Body composition was assessed using dual‐energy X‐ray absorptiometry (DXA) (Lunar iDXA, EnCore 2008 software version 12.20, GE Healthcare, WI) by professionals trained and supervised by a certified clinical densitometrist. For those participants who did not fit into the scanning area, a half‐body analysis was performed to estimate total body measurements of the right side using the mirror imaging tool [33]. Fat free mass (FFM) and FM were obtained, which was also expressed in terms relative to body mass (%FM). The visceral adipose tissue (VAT) was obtained using CoreScan VAT software, as previously described [34].

### 2.7 Hormonal Profile

Fasting blood samples were collected by a qualified professional. Serum insulin, cortisol, TSH, free T3, and free T4 were analyzed in refrigerated samples on the same day of blood collection by automated chemiluminescent immunoassay at the Clinical Analysis Laboratory (Cápsula/UERJ). Aliquots of serum were stored (−80°C) until additional analyses. Serum concentrations of total GLP‐1, leptin, ghrelin and adiponectin, were determined by enzyme‐linked immunosorbent assay (ELISA), using commercial kits (Merk S.A). Samples from the same participant were analyzed in the same assay batch. Intra and inter‐assay coefficients of variation were < 7% and < 10%, respectively.

### 2.8 Appetite Feelings

Participants assessed their desire to eat, hunger, satiety and prospective food consumption (PFC) according to 4 visual analogue scales (VAS) measuring 10 cm, without tick marks. The assessment was conducted before, immediately after, and 60 min after consuming the same typical and standardized breakfast (300 kcal; 17.4% protein, 28.3% fat, 54.3% carbohydrate), consisting of bread, cheese, and orange juice. The meal corresponded to approximately 20% of the average energy prescription for weight loss in the study [35]. Total area under the curve (AUC) for appetite feelings, both at baseline and after a week, was obtained considering the three testing time points (fasting, postprandial, and 60 min after the meal). AUC was calculated using the trapezoidal rule.

### 2.9 Statistics

Data analysis was conducted using an intention‐to‐treat approach. Summary measures of outcomes and covariates of interest were described as median and interquartile range (IQR) or mean and standard deviation (SD) according to the variable distribution. Differences between continuous independent variables were assessed using the *Student's t test* or Mann Whitney test. Differences between categorical variables were assessed using the Chi‐square test.

The effects of time, group, and their interaction, on body composition, hormonal profile, and appetite feelings were evaluated using linear mixed‐effects models. Each model was adjusted by including the baseline value of the respective outcome as a covariate. Multiple comparisons were adjusted using the Bonferroni post‐hoc comparisons. Analyses were also tested after adjustments for ‘body mass variation’ and ‘percentage of adherence’.

Differences between groups in RMR after 1 week were tested following the standardized ANCOVA framework proposed by Fernández‑Verdejo et al. (2026) [36]. (1) Model specification and diagnostics: A bidirectional stepwise procedure identified baseline RMR, fat‐free mass (FFM), and fat mass (FM) at week 1 as covariates. The ANCOVA primary model was: RMR_week1_ (kcal/day) ~ RMR_baseline_ + FFM _week1_ + FM _week1_ + Group. Residual normality and homoscedasticity were tested and indicated adequate model fit. Multicollinearity was examined via variance inflation factors (all < 5). (2) Interaction testing: Group × FFM_week1_ and Group × FM_week1_ interactions were tested and were not significant. (3) Visualization**:** Partial residuals were computed to visualize the adjusted association between FFM_week1_ and RMR_week1_. Median values of FM_week1_ and baseline RMR were fixed. Individual data points were plotted alongside model‐predicted regression lines for each group, reflecting ANCOVA coefficients (slope = β_FFM; vertical separation = β_Group). An additional ANCOVA was performed for ΔRMR (adjusted for baseline RMR, ΔFFM, and ΔFM), following the same procedures. *p* values < 0.05 were considered significant. Statistical analysis was performed with RStudio (version: 2024).

## 3 Results

### 3.1 Baseline Characteristics

**Figure 1:**
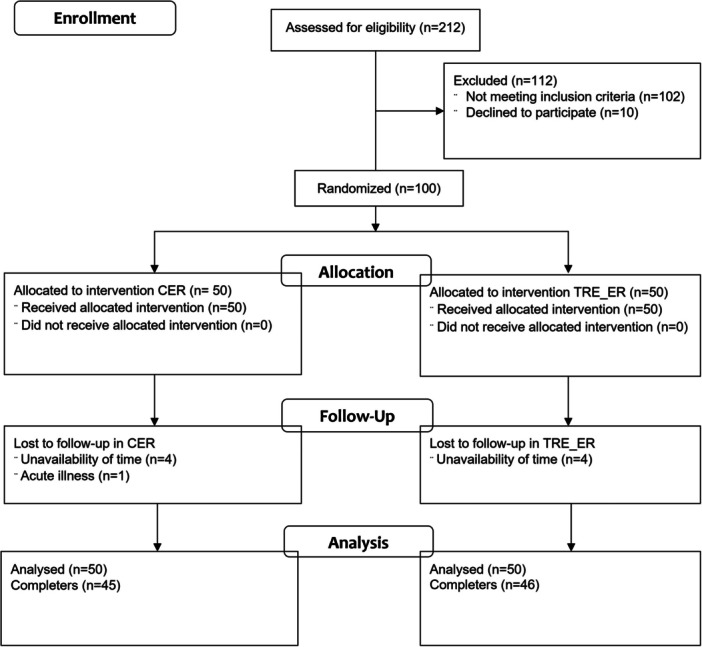
CONSORT diagram of the intervention.

A total of 100 adults were randomized between 2022 and 2024, and all were included in the analyses (*n* = 50 in TRE + ER) (Figure 1). Their mean age was ~33 years and BMI was ~44 kg/m^2^. The prevalence of diabetes (38% in the CER and 28% in the TRE + ER, *p* = 0.36) and hypertension (42% in the CER and 30% in the TRE + ER, *p* = 0.30) was not significantly different between groups. There were no significant differences between groups in terms of age, BMI, TEE, TEE_60%_, usual daily energy intake, or usual eating window (*p* > 0.30). Likewise, physical activity levels determined by IPAQ were similar between groups (*p* = 0.85; Table 1). RMRm and RMRp was also similar between groups. No differences were found between RMRp and RMRm in both sexes (Supporting Table S1). The interventions were carried out for 7 d (CER, IQR = 5 d and TRE + ER, IQR = 2 d). The percentage of adherence was 46 ± 72% in CER and 28 ± 44% in TRE + ER, with no significant difference between groups (*p* = 0.26) (Supporting Table S2).

**Table 1:** Characteristics of individuals with severe obesity at baseline ‐ MACRO study.

Variables	CER	TRE + ER	*p*‐value
	*n* = 50	*n* = 50	
Age (years)	33 (13)	32 (10)	0.735
Sex (W/M)	39/11	39/11	1.000
Comorbidities (*n* %)			
Diabetes type 2	19 (38)	14 (28)	0.355
Hypertension	21 (42)	15 (30)	0.298
Physical activity level (*n* %)			
Inactive	15 (30)	13 (26)	0.848
Irregularly active B	8 (16)	8 (16)	
Irregularly active A	11 (22)	11 (22)	
Active	12 (24)	16 (32)	
Very active	4 (8)	2 (4)	
BMI (kg/m^2^)	44.4 (8.44)	43.3 (5.72)	0.080
TEE (kcal/d)	2360 (646)	2284 (908)	0.975
TEE_60%_ (kcal/d)	1416 (387)	1370 (545)	0.959
Usual energy intake (kcal/d)	2035 ± 119	1926 ± 91.6	0.468
Usual eating window (h)	10.7 ± 0.33	11.1 ± 0.29	0.282

*Note:* Values are *n* (%) for categorical variables; median (IQR) and mean ± SD for continuous variables. CER: continuous energy restriction; TRE + ER: time‐restricted eating combined with energy restriction; TEE: total energy expenditure; TEE_60%_: dietary prescription corresponding to 60% of the total energy expenditure (TEE).

### 3.2 Body Composition

After adjustment for baseline values, no significant group effect was observed on body composition measures, except for FFM (Table 2). In the overall sample, significant time effects were observed, with decreases in all body composition measures (*p* < 0.01), except for %FM. The magnitude of reduction in FFM was higher in the TRE + ER group (TRE + ER ~ −800 g *vs*. CER ~ ‐100 g; group x time interaction, *p* = 0.032).

**Table 2:** Effect of a one‐week time‐restricted eating combined with energy restriction on body composition, appetite ratings and hormonal profile in adults with severe obesity ‐ MACRO study.

	T0		T1				
Variables	CER	TRE + ER	CER	TRE + ER	*p*‐value		
	*n* = 50	*n* = 50	*n* = 45	*n* = 46	Group	Time	Group* time
Body mass (kg)	123.9 ± 0.2	123.9 ± 0.2	123.4 ± 0.2^a^	122.8 ± 0.2*,^a^	0.104	< 0.001	0.119
FM (kg)	62.9 ± 0.13	62.9 ± 0.13	62.6 ± 0.14	62.5 ± 0.14^a^	0.808	0.006	0.921
%FM	50.8 ± 0.08	50.8 ± 0.08	50.7 ± 0.08	50.9 ± 0.08	0.299	0.603	0.264
FFM (kg)	60.9 ± 0.13	61.0 ± 0.13	60.8 ± 0.13	60.2 ± 0.13*,^a^	0.042	< 0.001	0.032
VAT (kg)	2.23 ± 0.02	2.23 ± 0.02	2.16 ± 0.03^a^	2.15 ± 0.03^a^	0.809	0.001	0.813
AUC hunger (cm* min)	122 ± 8.84	124 ± 8.93	146 ± 9.49	110 ± 10.3*	0.067	0.573	0.048
AUC desire to eat (cm* min)	151 ± 8.97	149 ± 9.07	141 ± 9.62	136 ± 10.4	0.727	0.240	0.857
AUC satiety (cm* min)	294 ± 12.5	294 ± 12.5	307 ± 13.5	330 ± 14.4	0.385	0.069	0.376
AUC PFC (cm* min)	168 ± 7.36	165 ± 7.36	145 ± 7.90^a^	155 ± 8.46	0.604	0.033	0.388
Insulin (μUI/mL)	22.0 ± 0.98	22.1 ± 0.98	20.1 ± 1.05	18.8 ± 1.04^a^	0.555	0.011	0.488
TSH (μUI/mL)	3.25 ± 0.10	3.18 ± 0.10	2.80 ± 0.11^a^	3.12 ± 0.11*	0.240	0.017	0.061
T3 (pg/mL)	3.22 ± 0.03	3.19 ± 0.03	3.19 ± 0.04	3.19 ± 0.04	0.682	0.624	0.820
T4 (ng/mL)	1.24 ± 0.02	1.24 ± 0.02	1.24 ± 0.02	1.23 ± 0.02	0.723	0.926	0.617
Cortisol (µg/dL)	12.4 ± 0.35	12.5 ± 0.34	12.2 ± 0.37	12.9 ± 0.36	0.294	0.698	0.432
GLP‐1 (pM)	43.6 ± 1.65	43.6 ± 1.61	43.6 ± 1.65	43.1 ± 1.61	0.883	0.902	0.877
Leptin (ng/mL)	49.5 ± 0.75	49.4 ± 0.75	47.4 ± 0.76^a^	45.9 ± 0.75^a^	0.278	< 0.001	0.366
Adiponectin (ng/mL)	16.8 ± 0.59	16.9 ± 0.58	16.1 ± 0.60	15.3 ± 0.58	0.562	0.058	0.427
Ghrelin (pg/mL)	578 ± 14.6	580 ± 14.4	584 ± 14.8	583 ± 14.6	0.955	0.738	0.909

*Note:* Values are estimated marginal means ± SE from linear mixed‐effects models adjusted for baseline values. FM: Fat mass; FFM: Fat free mass; RMR: resting metabolic rate; RMRp: resting metabolic rate derived from equation using baseline data; RQ: respiratory quotient; AUC: total area under the curve; PFC: prospective food consumption. GLP‐1: glucagon‐like peptide‐1. *P* values refer to each component of the interaction model (intervention group, time, and group*time interaction term), in the linear mixed‐effects model.

* Indicates significant difference from CER group at the same time (Bonferroni post‐hoc analysis; *p* < 0.05).

^a^ Indicates significant difference from T0 within the same group (Bonferroni post‐hoc analysis; *p* < 0.05).

### 3.3 Hormonal Profile and Appetite Feelings

No significant group effect was observed on hormonal profile and appetite feelings (Table 2). Statistically significant decreases over time were observed in serum concentrations of insulin (~ 12%, *p* = 0.011), leptin (~ 6%; *p* < 0.001) and TSH (~ 8%; *p* = 0.017). No time effect was observed for T3, T4, cortisol, GLP1, adiponectin and ghrelin. There was no group x time interaction detected in the hormonal profile, except for TSH concentrations that approached statistical significancy (*p* = 0.061). TSH decreased significantly only in the CER group (*p* = 0.003), which lead to lower concentrations in the CER group compared to TRE + ER after 1 week (Bonferroni post‐hoc *p* = 0.036).

Statistically significant decreases over time were observed in AUC of PFC (~ −17 cm*min; time‐effect *p* = 0.033). AUC of hunger increased in the CER group (24 cm*min) and decreased in the TRE + ER (−14 cm*min; group x time interaction, *p* = 0.048) (Table 2). The effect of group, time, and group x time interaction was also tested after adjusting for ‘body mass variation’ and ‘% adherence’ with no alterations on the results for body composition measures, resting metabolism, appetite feelings and hormonal profile (data not shown).

### 3.4 RMR and Early Adaptive Thermogenesis

**Figure 2:**
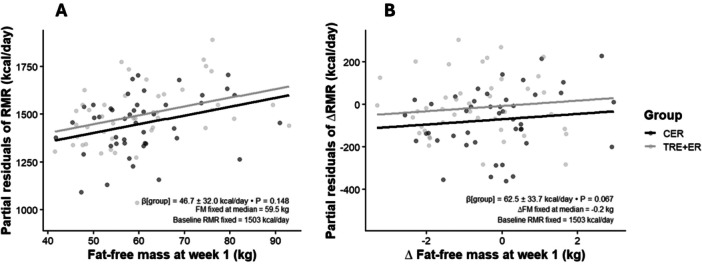
Group effect on resting metabolic rate (RMR) and RMR change. (A) Resting metabolic rate after one week of intervention, adjusted for baseline RMR, fat‐free mass and fat mass at week 1. (B) Change in RMR from baseline (ΔRMR), adjusted for change in fat‐free mass and fat mass. The plot displays partial residuals and model‐derived regression lines, reflecting the adjusted group effect on RMR, as proposed by Fernandez‐Verdejo et al., 2026 [36].

RMR at week 1 did not differ between groups after adjustment for baseline RMR, FFM _week1_, and FM _week1_ (β_group = 47 ± 32 kcal/day; *p* = 0.148) (Figure 2A). Similarly, changes in RMR, adjusted for baseline RMR and changes in FFM and FM, did not differ between groups. However, the TRE + ER group showed a trend toward a lower reduction in RMR (β_group = 63 ± 34 kcal/day; *p* = 0.067) Figure 2B). Unadjusted comparisons of ΔRMR showed a mean change of −0.27 ± 155 kcal/day in the TRE + ER group and −85 ± 151 kcal/day in the CER group (*p* = 0.07; Supporting Figure S1).

**Figure 3:**
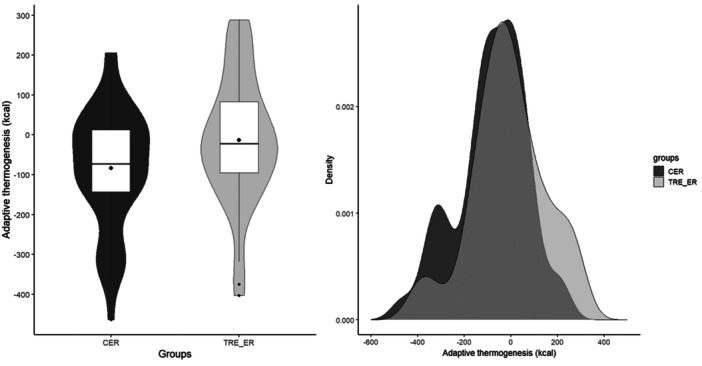
The early adaptive thermogenesis (calculated according to Nunes et al. [32]) distribution in continuous energy restriction (CER) and time‐restricted eating combined with energy restriction (TRE + ER) group. The diamond indicates the mean adaptive thermogenesis for the TRE + ER and CER groups (−13 ± 160 kcal *vs*. −83 ± 150 kcal; *p* = 0.038).

Mean early AT values, calculated according to Nunes et al. [32], were significantly lower in the TRE + ER compared to the CER group (−13 ± 160 kcal *vs*. −83 ± 150 kcal; *p* = 0.038) (Figure 3). Early AT (i.e., negative values) was observed in 68% of CER participants and 52% of TRE + ER participants, with no significant difference between groups (chi‐square; *p* = 0.191).

## 4 Discussion

We herein reported the effects of TRE on metabolic adaptation during energy restriction in the first phase of weight loss in individuals with severe obesity. After 1 week of a prescribed energy restriction diet corresponding to 60% of TEE, RMR was similar between groups when adjusted for body composition. Nevertheless, our results suggest that TRE promoted a subtle mitigation of AT. Both interventions led to expected decreases in insulin, leptin and TSH serum concentrations. The feeling of hunger increased after 1 week only in the CER group.

It has been suggested that AT after 1 week of energy restriction, i.e. early AT, is an important predictor of subsequent weight loss [8, 9], and therefore should be considered for managing obesity. Early AT has been observed at various levels of energy expenditure, including TEE (−178 ± 137 kcal/d), sleep energy expenditure (−101 ± 145 kcal/d) [9], and RMR (−65 ± 198 kcal/d) [7]. Notably, when assessing TEE, RMR is suggested to be the component undergoing early AT [1]. At the level of RMR, there is evidence that AT occurs in up to 60% of individuals undergoing energy restriction [7]. Consistent with previous findings, we observed early AT in the majority (CER = 68%; TRE + ER = 52%) of individuals with severe obesity in this study.

The effects of TRE on both early and long‐term AT have not been studied, although several investigations have assessed its effects on energy metabolism components [20, 37, 38, 39, 40]. In the present study, the magnitude of AT was slightly lower in the TRE group, suggesting a limited effect of TRE on mitigating AT. It has been shown that, when combined with energy restriction, TRE did not appear to affect RMR in women with overweight [39], whereas in women with obesity TRE resulted in a trend toward increased RMR [38]. In the present study in individuals with severe obesity, after 1 week of intervention, a non‐significant trend toward lower decline in RMR was observed in the TRE group based on unadjusted data. Indeed, no between‐group differences were detected when RMR was adjusted for body composition, as recommended [36]. Together, these findings suggest that, although early AT may be modestly attenuated by TRE, this effect is unlikely to result in meaningful differences in energy expenditure beyond changes in body composition in the short term.

Besides de metabolic adaptation at the level of RMR, the early phase of weight loss is also characterized by hormonal adaptations that affect glycolytic and oxidative activity of metabolically active organs, causing metabolic deceleration [7]. The reduction in insulin secretion promotes glycogen depletion and losses of intracellular and extracellular water, which partly explain the initial decline in fat‐free mass (FFM) [1, 6]. In the present study, the greater reduction in FFM observed in the TRE + ER group after 1 week may therefore reflect more pronounced short‐term changes in glycogen stores and hydration status [41], possibly related to the extended fasting window, rather than true lean tissue loss. It has been demonstrated that during the first week of energy restriction, thyroid hormones and leptin also decrease alongside insulin levels [1, 7, 9], as also observed in the present study. No additional hormonal changes were detected. However, it is important to note that only fasting hormone concentrations were assessed; therefore, potential effects of TRE on postprandial gut hormones, such as GLP‐1, cannot be excluded.

Appetite feelings were not assessed during the first week of energy restriction in previous studies. Our results indicate that TRE may mitigate the expected increase in hunger during the early phase of energy restriction. However, in longer interventions, no effect of TRE on appetite feelings has been observed [25, 38, 42].

This study has several strengths. It was randomized and controlled, ensuring that the groups had similar characteristics at the beginning and were subjected to the same level of energy restriction. Furthermore, the study was conducted in a growing population of individuals with severe obesity, a group for which there is limited research on body composition, energy expenditure, appetite, and hormonal profiles during dietary interventions. To our knowledge, this is the first study to evaluate the effects of TRE + ER on early metabolic adaptation.

This study also has limitations. The short intervention period, inherent to the objective of evaluating early AT, was challenging due to subtle changes in body composition and RMR. As DXA does not capture changes in water content or organ mass, accuracy of RMR estimation may be compromised, potentially introducing errors in AT calculation and interpretation [1, 4]. Future studies on early adaptations to energy restriction should incorporate methods capable of assessing changes in body water compartments, such as isotope dilution, together with imaging techniques such as computed tomography and magnetic resonance imaging to provide a more reliable characterization of short‐term changes in FFM [43, 44].

Nevertheless, in the present study, RMR was also analyzed at week 1 adjusted for concurrent body composition, which likely mitigates, at least in part, the impact of DXA‐related measurement error. We cannot exclude the possibility that part of the RMR change over 1 week is due to inherent calorimetry measurement error, potentially influencing results interpretation. However, we believe that this potential error was similar in both groups. In addition, in the present study, AT was assessed only at the level of RMR. Although early AT appears to be particularly relevant at this level during the initial phase of weight loss [1], future studies should also assess non‐resting energy expenditure using methods such as doubly labeled water or whole‐room indirect calorimetry. Another limitation was the low adherence to the prescribed energy restriction observed in both groups, possibly due to the short intervention period and the participants' routine adaptation to the new prescribed diet, which may have hampered the evaluation of the effects of TRE under energy restriction. However, the energy restriction actually achieved (~ 85% of TEE) was sufficient to induce significant and similar reductions in body mass in both groups during the intervention period. Finally, although standardized meals are commonly used in studies assessing appetite feelings [3, 5, 45], they may provide different magnitudes of hormonal and appetite‐related stimuli depending on the individual's energy requirements. However, because both sex distribution and mean TEE were similar between groups, we believe the impact of this interindividual variability was minimal.

## 5 Conclusion

We observed early AT in more than half of individuals with severe obesity, with only a modest mitigating effect of TRE. Overall, metabolic adaptation during the initial phase of weight loss—including early AT, resting metabolic rate, hormonal changes, and appetite responses—does not appear to be substantially modified by TRE. Future studies using sensitive methods to assess FFM are needed to clarify the determinants of variability in early metabolic adaptation in this population. Further research should also examine whether the modest early effects of TRE translate into clinically meaningful long‐term benefits for weight management.

## Author Contributions

T.A.M.C. and Flávia Fioruci Bezerra conceived the study. T.A.M. and Raiane Lira Freitas Brasil collected the data. T.A.M. conducted the analysis and drafted the manuscript. All authors critically revised the manuscript and approved the final version.

## Conflicts of Interest

The authors declare no conflicts of interest.

## Supporting information


**Figure S1:** RMR change after one week distribution in continuous energy restriction (CER) and time‐restricted eating combined with energy restriction (TRE+ER) group. The diamond indicates the mean of RMR change after one week for the TRE+ER and CER groups (raw comparison: −85 ± 151 kcal vs. −0.27 ± 155 kcal; P = 0.07; Student's T test).
**Table S1:** Measured and predicted resting metabolic rate at baseline.
**Table S2:** The expected daily energy lost, and actual daily energy lost after one week of energy restriction.

## Data Availability

The data that support the findings of this study are available from the corresponding author upon reasonable request.
