# Supplementary material for: Effect of Time‐Restricted Eating on Metabolic Adaptation in Adults With Severe Obesity During Early Phase of Weight Loss
Source: J Hum Nutr Diet. 2026 Jun 11;39(3):e70293. doi: 10.1111/jhn.70293 (PMC13260697; doi:10.1111/jhn.70293)
Supplement: Supplementary file 1 — Figure S1: RMR change after one week distribution in continuous energy restriction (CER) and time‐restricted eating combined with energy restriction (TRE+ER) group. The diamond indicates the mean of RMR change after one week for the TRE+ER and CER groups (raw comparison: −85 ± 151 kcal vs. −0.27 ± 155 kcal; P = 0.07; Student's T test). Table S1: Measured and predicted resting metabolic rate at baseline. Table S2: The expected daily energy lost, and actual daily energy lost after one week of energy restriction. [file JHN-39-0-s001.docx]

**Supplementary material**

**Effect of time-restricted eating on metabolic adaptation in adults with severe obesity during early phase of weight loss**

Table S1. Measured and predicted resting metabolic rate at baseline.

|  | CER | | TRE+ER | |
| --- | --- | --- | --- | --- |
|  | Women  (n= 39) | Men  (n=11) | Women  (n=39) | Men  (n= 11) |
| Baseline RMR measured (kcal/d) | 1521 ± 198 | 1985 ± 316 | 1486 ± 196 | 1985 ± 189 |
| Baseline RMR predicted (kcal/d) | 1503 ± 94.4 | 1914 ± 145 | 1508 ± 132 | 1945± 140 |

CER: continuous energy restriction. TRE+ER: time-restricted eating combined with energy restriction. No difference was observed between groups (t-test for independent samples; p > 0.05) or between measured and predicted RMR (t-test for paired samples; p > 0.05) for both sexes.

Table S2. The expected daily energy lost, and actual daily energy lost after one week of energy restriction.

|  | CER | TRE+ER | p-value |
| --- | --- | --- | --- |
| Expected daily energy lost (kcal) | 805 ± 359 | 874 ± 354 | 0.361 |
| Actual daily energy lost (kcal) | 209 ± 623 | 126 ± 464 | 0.481 |
| Percentage of adherence (%) | 46 ± 72 | 28 ± 44 | 0.161 |

CER: continuous energy restriction. TRE+ER: time-restricted eating combined with energy restriction. Expected daily energy lost: daily total energy expenditure - energy prescribed. The actual daily energy lost was calculated according to Del Corral et al., 2009 [29].


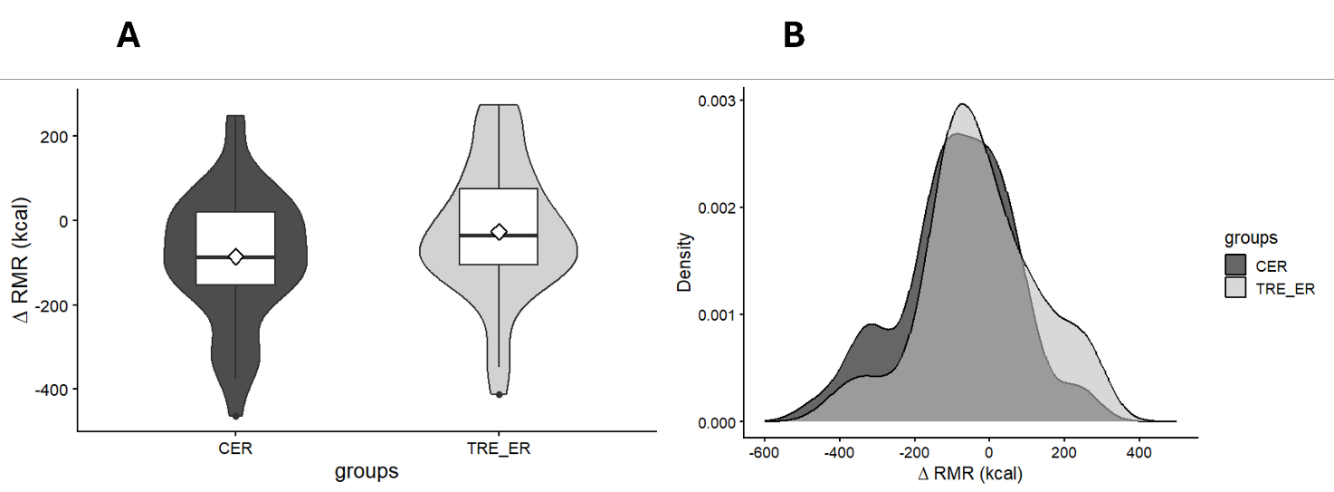


Figure S1. RMR change after one week distribution in continuous energy restriction (CER) and time-restricted eating combined with energy restriction (TRE+ER) group. The diamond indicates the mean of RMR change after one week for the TRE+ER and CER groups (raw comparison: -85 ± 151 kcal *vs*. -0.27 ± 155 kcal; P = 0.07; Student´s T test).
